# Efficacy and safety of polyherbal formulation as an add-on to standard-of-care in mild-to-moderate COVID-19: A randomized, double-blind, placebo-controlled trial

**DOI:** 10.1016/j.jaim.2022.100653

**Published:** 2022-10-24

**Authors:** Suresh B. Patankar, Anupama Gorde, Kalpana Joshi, Kishor Suryawanshi, Pravin Soni, Tejas Shah, Sagar Patankar, Diwakar Jha, Rajesh Raje, Hrishikesh Rangnekar

**Affiliations:** a AMAI Charitable Trust, Pune, India; b Arogyasewa Medical Academy of India Trust, Pune 411 052, MS, India; c Sinhgad Institute of Medical Sciences, Pune, India; d Dept. of Biotechnology, Sinhagad College of Engineering, Pune, India; e YCM Hospital, Pune, India; f KRSNNA Diagnostics Pvt. Ltd., Pune, India; g SHRIPAD Medisearch Pvt. Ltd. Pune, India; h Ayurvedic Physician, Quest Clinical Services, Pune, India

**Keywords:** COVID-19, Herbal medication, Viral load, Immunomodulation

## Abstract

**Background:**

Novel corona virus disease-2019 (COVID-19) pandemic is a significant contributor to morbidity and mortality in affected individuals. Modulating the immune response in COVID-19 is now an established treatment approach. Polyherbal formulations have long been assessed for their potential immune modulating effects and are expected to be beneficial on COVID-19.

**Methods:**

This study aims at assessing the efficacy and safety of polyherbal formulation (referred as IP) in comparison to placebo, as add on to the standard of care (SOC), in patients with mild to moderate COVID-19 patients. Hospitalized RT-PCR positive patients were randomized to either SOC + IP or SOC + Placebo arm. The viral load (VL) was assessed using quantitative reverse transcription-polymerase chain reaction (qRT-PCR). Immunological parameters were also assessed. The clinical improvement was assessed using a numeric rating scale (NRS) and WHO ordinal scale, and follow-up period was 30 days.

**Results:**

Seventy-two patients were randomized to SOC + IP (n = 39) and SOC + Placebo (n = 33) arms. There was significant reduction in VL in SOC + IP arm from day 0–4 (p = 0.002), compared to SOC + Placebo arm (p = 0.106). Change in the NRS score and WHO score was significant in both arms, however, the difference between the two arms was statistically significant in favour of IP arm. The increase in Th1 response was significant in SOC + IP arm (p = 0.023), but not in SOC + Placebo arm. COVID-19 specific antibodies were numerically higher in the SOC + IP arm.

**Conclusion:**

The study finds that polyherbal formulation significantly reduces VL and contributes to immunomodulation and improvement in clinical conditions without side effects.

## 1 Introduction

Novel corona virus disease 2019 (COVID-19) pandemic is a significant contributor to morbidity and mortality in affected individuals. World Health Organization, as of 23 April 2021, reports 144,358,956 confirmed cases of COVID-19, including 3,066,113 deaths [1]. Since its first identification in December 2019, there is now a fair understanding of the disease pathogenesis. Hyper stimulated immune response causing systemic inflammatory response syndrome or ‘cytokine storm’ is known to be implicated in progression to severe COVID-19 [2,3]. Such immune response correlates linearly with the viral load that in turn correlates with disease severity [4,5]. Modulating the immune response in COVID-19 is now an established treatment approach. The RECOVERY trial showed that Dexamethasone 6 mg, once daily for ten days, significantly lowered 28-day mortality in hospitalized COVID-19 patients [6]. Also, treatment with specific interleukin-6 inhibitor Tocilizumab did not improve survival, but reduced the likelihood of progression to the composite outcome of mechanical ventilation or death [7]. With Itolizumab, an anti-CD6 humanized monoclonal antibody with an immunomodulating action on T effector cells, there was a significant reduction in 30-day mortality along with significant improvement in clinical immunological, and oxygen parameters [8]. Thus, modulating immune response can help to improve clinical parameters and mortality outcomes, especially in hospitalized moderate to severe COVID-19 patients.

Herbal formulations have long been assessed for their potential immune modulating effects [9,10]. In an in-vitro study (data on file), we observed that a combination of herbals (IP) such as Ashwagandha (*Withania somnifera*), Vidanga (*Embelia ribes*), Guduchi (*Tinospora cordifolia*), Haritaki (*Terminalia chebula*), Aamalaki (Emblica officinalis), Shatavari (*Asparagus racemosus*), Yeshtimadhu (*Glycyrrhiza glabra*), Ginger (Zinziber officinale), Pippali (*Piper longum*), Shankha Bhasma and Jasad Bhasma showed antiviral activity against SARS-CoV-2. As the search for potential antiviral and immune modulating therapy for COVID-19 is ongoing, this could be potentially beneficial. Therefore, we conducted a randomized, double-blind, placebo-controlled study to determine the effect of polyherbal formulation on viral load, immunological parameters and clinical improvement in mild to moderate COVID-19 disease, when used as an add-on to the SOC. The primary aim of the study was to determine the: a) change in viral load, b) change in disease severity score, clinical improvement in respiratory health, and c) change in immunological and inflammatory markers.

## 2 Methods

### 2.1 Design and setting of the study

This study was a double-blind, randomized, placebo-controlled trial to assess the safety and efficacy of polyherbal drug formulation (designated as IP) in patients with mild to moderate COVID-19. The study was conducted at a single center in Pune, Maharashtra, India. We enrolled adults of age 18 years and above, diagnosed with laboratory-proven COVID-19 disease of mild to moderate severity, who were admitted to the hospital as per the isolation protocol. Numeric rating scale (NRS) was used for overall disease severity assessment. Normal status was considered as 0, while 1–3 was referred as mild, 4–6 as moderate and 7–10 as severe disease condition.

Patients with uncomplicated upper respiratory tract infection, with symptoms such as fever, cough, sore throat, nasal congestion, malaise, headache without evidence of breathlessness or Hypoxia (SpO 2 < 94) were considered as mild; and pneumonia with no signs of severe disease were considered as moderate according to Clinical Management Protocol: COVID-19, by GOI. Ministry of Health and Family Welfare (Version 3 13.06.20). NRS rating was used for assessment of clinical improvement. Majority part of NRS was occupied by Respiratory Symptoms (cough and breathlessness), and hence NRS can be extrapolated to Respiratory Health to a great extent. Besides cough, anosmia was present in mild cases whereas SpO2 < 94% and/or radiologic/clinical evidence of lower respiratory tract disease were considered for labelling moderate disease. Later for analysis, Novel Corona COVID-19 Therapeutic Trial Synopsis by WHO was used for ordinal rating scale.

Pregnant and lactating females, any respiratory symptoms of >7 days, patients with known pathology affecting the respiratory system, diagnosed hematological disorders, patients with a terminal illness, any other condition, which in view of the investigator would interfere with the general clinical well-being of the participant, and those not willing to participate in the study were excluded. The trial was conducted as per the ethical principles of the Declaration of Helsinki, good clinical practice recommendations and applicable local regulatory guidelines. Informed consent was obtained from all the participants before enrolment. Study period was between August 2020 till October 2020. The study was registered with the clinical trial registry of India (CTRI/2020/07/026570). The study protocol was published in a peer-reviewed journal [11].

### 2.2 Characteristics of participants

Patients with a known positive status of RT-PCR for SARS-CoV-2 disease were screened for the study. Eligible and consenting patients were enrolled. Patients were randomized using computer-generated randomization codes to receive either IP or Placebo as an add-on to the recommended standard care (SOC) for COVID-19, as per the protocol prescribed then by the Indian Council of Medical Research (ICMR) following WHO guidelines. All the patients continued their routine diet, physical activity and other prescribed treatment including antiviral drugs.

### 2.3 Processes, interventions and comparisons

#### 2.3.1 Investigational product (IP)

The investigational products were manufactured per API guidelines and as per guidelines for Randomized, Controlled Trials of Herbal Interventions. The scientific details for both the IPs are given in Table 1S. The placebo capsule**,** filled with lactose powder, used in trial were of identical size and color so as to match with the IP capsule with coding known only to sponsor were supplied in identical container bottles. Patients were advised to take one capsule, two times a day (of each IP1 and IP2) or placebo, after meals. The dose of IP1 was 400 mg, while IP2 was 450 mg. Patients received a prepacked carton of study treatment for a total duration of 30 days. Capsule IP1 and IP2 were given for the first 15 days, and last 15 days they were given only IP2. Patients receiving placebo also received 2 capsules twice a day for first 15 days and for next 15 days, one capsule twice a day.

During baseline visit (Day 0), nasopharyngeal swab was taken for qRTPCR, NRS was filled for clinical status and the blood was withdrawn for various immunological, inflammatory and safety markers. Patients had three follow-ups during the 30-day treatment period. First follow-up visit was done at day 4 ± 1. qRTPCR and NRS were repeated at this visit and any clinical adverse events were recorded. Second follow-up was done clinically or telephonically (if they were discharged) on day 15 ± 2 to record any clinical deterioration in clinical condition and to record adverse effects, if any. Last follow-up was at day 30 ± 2 to document clinical condition, adverse effects and perform laboratory investigations. In this last follow-up, study personnel visited patients in their homes to avoid their repeat visit to site, as it was an active COVID-19 hospital. Patients were asked to visit any time, if they observed any deterioration in clinical condition or had developed any untoward reactions.

The quantitative estimation of viral load was carried out using qRTPCR. The RNA extractions were carried out on ThermoFischer Scientifics’ KingFisher™ Flex Purification System using the MagMAX™ Viral/Pathogen II (MVP II) Nucleic Acid Isolation Kit. The RT-PCR was carried out on Qiagen Rotorgene Q- PCR system using ICMR approved GenePath CoViDx One RT-qPCR v 2.1.1 assay. The method was approved by Indian Council of Medical Research (ICMR). The method has been validated extensively using synthetic SARS-CoV-2 RNA control with known concentrations from Twist Bioscience and synthetic SARS-CoV-2 viral particles from BEI Resources.

### 2.4 Sample collection and nucleic acid extraction

Clinical samples for SARS-CoV-2 testing consisted of nasopharyngeal and oropharyngeal swabs collected from each patient and transported in Viral Transport Medium (VTM). After receiving the samples in the laboratory, the tubes were vortexed and centrifuged at 2500 rpm for 2 min. Two hundred microlitres of the sample was used as input in Thermo Fisher Scientifics’ KingFisher™ Flex Purification System using the MagMAX™ Viral/Pathogen II (MVP II) Nucleic Acid Isolation Kit. The RNA was eluted in a 50uL elution buffer and used for downstream processing.

Three viral loci RdRP, N gene and E gene were used to estimate viral load using GenePath Diagnostics’ indigenously developed qRTPCR assay, CoViDx One v2.1.1 keeping appropriate controls and standards. Ten microlitres of extracted nucleic acid was used as an input for the qRT-PCR. Quantitative estimation for each genomic target was performed using standards, for which the number of genome copies were already known. The threshold cycle (Ct value) for each genomic target was extrapolated to the viral load of standards to estimate the number of copies/uL, which were then converted to copies/mL for each gene for each sample. Average number of copies/ml was calculated for each sample that accounts for the approximate viral load in the sample. Ct values for control human gene (RNaseP) ensured the adequacy of sample, nucleic acid extraction and nucleic acid amplification.

On the day of first encounter with patients, they were subjected to thorough clinical and laboratory investigations. After signing of informed consent, their demographic details, and clinical parameters were recorded. Oxygen saturation was assessed using pulse oximeter. Blood was drawn for lymphocyte subset analysis (absolute neutrophil count, absolute lymphocyte count, TH1, TH2, Treg cells, NK Cells and CD markers such as CD3, CD4, CD3: CD8 ratio) as well as serum IgG and IgM levels (not specific to COVID-19). Liver function tests and renal function tests were performed as a part of safety assessment. Blood investigations were repeated on day 30 for immunological markers, and for safety assessments. We performed all the tests at a single accredited laboratory.

### 2.5 Outcome measures

Primary efficacy outcome measure was change in viral load. Secondary efficacy outcome measures were change in disease severity score, clinical improvement (improvement in respiratory health) and change in immunological and inflammatory markers. NRS was used for overall disease severity as mentioned above. Patients were asked to rate the disease severity on the NRS. WHO ordinal Score was used for ordinal rating scale. Also, change in immunological parameters such as lymphocyte subset analysis (TH1, TH2, Th17), NK Cells and CD markers, serum IgG and IgM levels and COVID-19 antibodies were assessed. Inflammatory and other markers like C- reactive protein and D-Dimer, Sr. Ferritin were assessed in a small portion of enrolled subjects. Primary safety measure was incidence of adverse reactions (clinical and/or laboratory parameters).

**Fig. 1:**
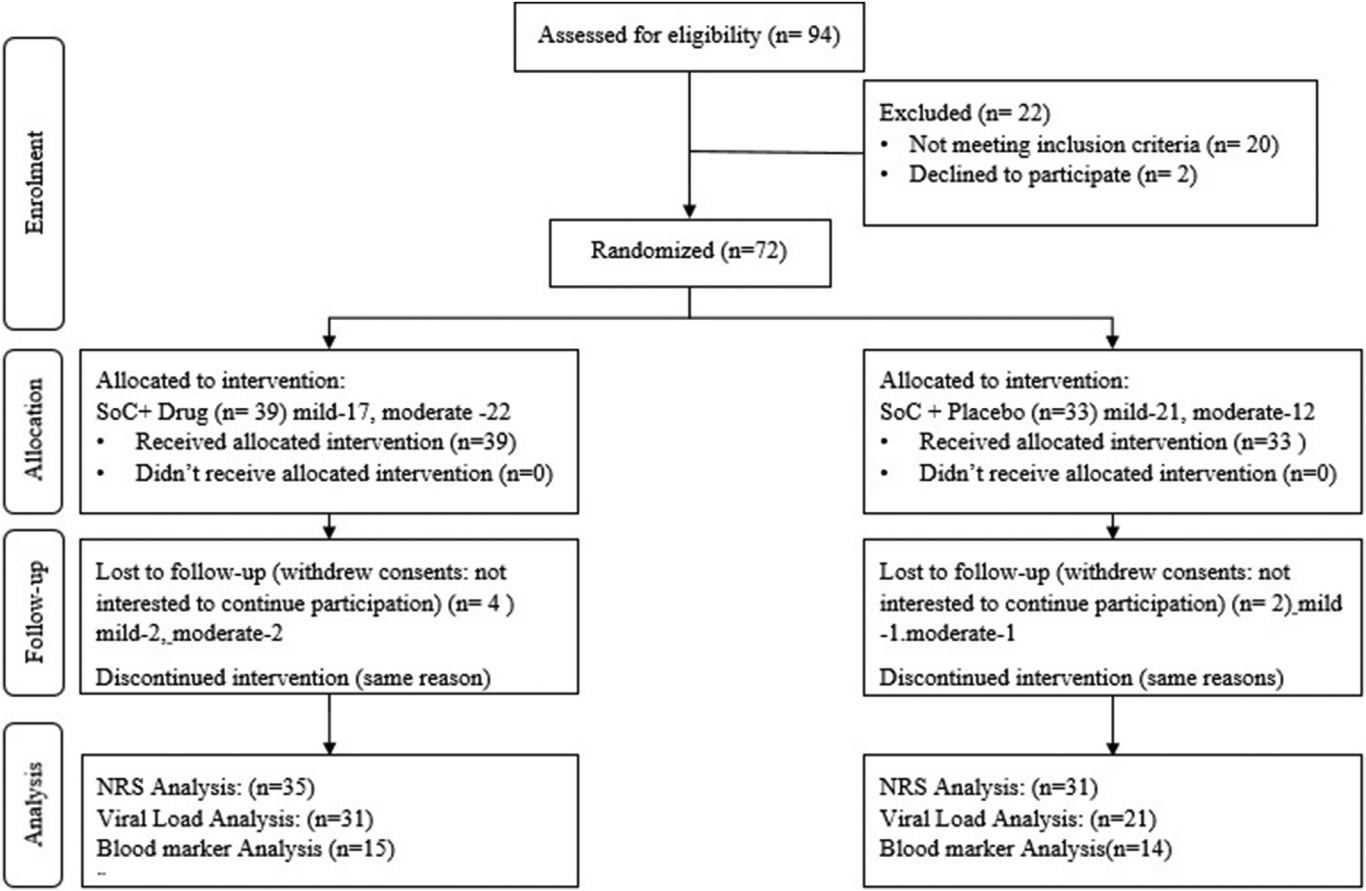
Study flow chart.

Regarding sample number of patients to be enrolled in the study, a total of 94 patients were screened, out of which 72 patients were eligible and randomized to two treatment arms, with 39 in SOC + IP group, while 33 to SOC + Placebo group. Fig. 1 shows the patient flow chart as per CONSORT guidelines. In SOP + IP group, 35 patients, while in SOC + Placebo, 31 patients completed the study.

### 2.6 Statistical analysis

Regarding sample size, the chances of outcome in the unexposed population was considered as 0.05, and in the exposed population was considered as 0.35. Further considering the odds ratio of 0.1 and the prevalence of 0.3, the estimated sample number of subjects per group with equal ratio was 28 (Total: 56), that can provide the risk estimate with 95% confidence and 80% power. Assuming 20% drop out rate, the final sample size per group was fixed to 35 patients.

Basic data on patient demography, anthropometry, and clinical investigations were captured according to scale of measurement. The bio-chemical investigations were performed according to study protocol at scheduled visits. The data was stored in a pre-designed Microsoft Excel 2016 worksheet for each patient according to visits. Validation checks were implemented time to time for data reliability and at study completion, the worksheet was locked. The subsequent statistical analyses were performed using SPSS Ver 20.0 (IBM Corp Armonk, New York USA) software.

Categorical variables were presented as frequency and percentage. Statistical comparisons of categorical data were done using Pearson's Chi-square test or Fischer exact test. For continuous variables, normality of data was assessed by referring to histograms. Normally distributed data was presented using mean and standard deviations (SD) and non-normally distributed data presented as median (Interquartile range (IQR) 25–75). Student t-test and Mann–Whitney U test were applied accordingly to test the statistical differences in continuous independent variables. For before and after analysis, we used paired t-test and Wilcoxon signed rank test to determine the statistical significance. A p-value < 0.05 was considered significant for all comparisons.

## 3 Results

### 3.1 Demographic parameters

The median age of patients in SOC + IP group was 47 years, while in SOC + Placebo group was 43 years; and the difference in the medians was statistically non-significant (p = 0.336) as shown in Table 1. There was no difference in proportion of males and females in two arms (p = 0.150). The socio-economic status of patients showed statistically non-significant difference between two groups (p = 0.905). In the SOC + IP arm, 3 (7.7%) subjects had DM, 5 (12.8%) subjects had HTN, while 2 (5.1%) had both DM & HTN. In the SOC + PL arm, 3 (9.1%) subjects had DM, 4 (12.1%) had HTN and 1 (3.0%) subject had both DM and HTN. The comorbidities present in both the groups were comparable at baseline. The mean BMI for patients in SOC + IP arm was 22.96 ± 4.01 kg/m^2^, while that of SOC + Placebo arm was 21.84 ± 3.54 kg/m^2^, and the difference between the two was statistically non-significant (p = 0.217). Regarding severity of disease, in the SOC + IP arm, 17 (43.6%) were mild, and 22 (56.4%) were moderate, while in the SOC + Placebo arm, 21 (63.6%) were mild, and 12 (36.4%) were moderate. The difference in the distribution of patients as per severity in two arms was statistically non-significant (p = 0.089). All the above characteristics showed statistically non-significant difference, thereby justifying the randomization.

**Table 1:** Descriptive statistics for various characteristics of patients in two groups.

Parameters	SOC + IP (n = 39)	SOC + Placebo (n = 33)	P-value
Age (years) [Median (IQR)]	47.0 (40.0–56.0)	43.0 (34.0–58.0)	0.336^a^
Gender [n (%)]			
Male	17 (43.6)	20 (60.6)	0.15^b^
Female	22 (56.4)	13 (39.4)	
Socio-economic status [n (%)]			
Upper middle	10 (25.6%)	7 (21.2%)	0.905^b^
Lower middle	17 (43.6%)	15 (45.5%)	
Upper lower	12 (30.8%)	11 (33.3%)	
Comorbidities [n (%)]			
Diabetes Mellitus only	3 (7.7%)	3 (9.1%)	0.894^b^
Hypertension only	5 (12.8%)	4 (12.1%)	
Both Diabetes and Hypertension	2 (5.1%)	1 (3.0%)	
Body mass Index (kg/m^2^) [Mean ± SD]	22.96 ± 4.01	21.84 ± 3.54	0.217^c^
Severity of disease [n (%)]			
Mild	17 (43.6%)	21 (63.6%)	0.089^b^
Moderate	22 (56.4%)	12 (36.4%)	

a Using Mann–Whitney U test.

b Using Pearson's Chi-square test.

c Using t-test for independent samples.

### 3.2 Effect on viral load (VL)

Overall, 52 patients had undergone qRT-PCR on day 4 (31 in SOC + IP, 21 in SOC + Placebo arm). Statistically significant reduction in viral load was observed in SOC + IP arm (The median changed from 662081 virus copies/mL (IQR: 56,904 to 19,485,712) on day 0–48963 copies/mL (IQR: 8774 to 1,248,257) day 4 with a p-value of p = 0.002 (Table 2). In SOC + Placebo arm, there was reduction in VL from median of 385670 copies/mL (IQR: 30,807 to 131,511,846) on day 0–66,845 copies/mL (IQR: 11,978 to 836124.5) on day 4; however, the difference was statistically insignificant (p = 0.106). The difference of medians at Day 4 between two treatment arms was statistically insignificant (p = 0.845).

**Table 2:** Comparison of viral load between and within groups.

Time	SOC + IP (n = 31) [Median (IQR)]	SOC + Placebo (n = 21) [Median (IQR)]	P-value^a^
Baseline	662,081 (56,904–19,485,712)	385,670 (30,807–131,511,846)	0.758
Day 4	48,963 (8774–1,248,257)	66,845 (11978–836124.5)	0.845
P value^b^	0.002	0.106	

a Using Mann–Whitney U test.

b Using Wilcoxon sign-rank test.

### 3.3 Clinical assessment

In the SOC + IP arm, there was a highly significant reduction in NRS from 4.3 ± 1.13 at day 0–1.74 ± 1.03 at day 4 (p < 0.0001) as shown in Table 3. In the SOC + Placebo arm as well, there was significant reduction in NRS score from 4.26 ± 1.09 to 3.16 ± 1.03 at day 4 (p < 0.0001). Baseline scores of both the arms were similar (p = 0.78); however, the difference between the two arms during the follow-up was statistically highly significant (p < 0.0001). The mean WHO score for the SOC + IP arm at baseline was 1.63 ± 0.84 and at follow-up was 1.03 ± 0.45, and the mean difference between the two was statistically highly significant (p < 0.0001). In the SOC + placebo arm, these values were 1.71 ± 0.78 at baseline and 1.26 ± 0.63 at follow-up, and the mean difference was statistically significant (p = 0.03).

**Table 3:** Comparison of NRS, WHO scores between and within groups.

NRS score	SOC + IP (n = 35) [Mean ± SD]	SOC + Placebo (n = 31) [Mean ± SD]	P value^a^
Baseline	4.3 ± 1.13	4.26 ± 1.09	0.781
Follow-up	1.74 ± 1.03	3.16 ± 1.03	<0.0001
P value	<0.0001	<0.0001	
WHO Ordinal Scale			
Baseline	1.63 ± 0.84	1.71 ± 0.78	0.58
Follow-up	1.03 ± 0.77	1.26 ± 0.63	0.33
P value^b^	<0.0001	0.03	

a Using t-test for independent samples.

b Using paired t test.

### 3.4 Blood biomarkers for immunity and inflammation

Changes in the immunological and inflammatory markers are shown in Table 4, between day 0 and day 30. There was significant increase in absolute B cell count, absolute T cell count, absolute CD3, CD4 and T helper cells as well as in the ratio of absolute CD3 and CD8 cells in both SOC + IP and SOC + Placebo arm (p < 0.05). Change in absolute NK cell count was significant in the SOC + Placebo arm (p = 0.020), but not in SOC + IP arm (p = 0.067). However, the magnitude of change was higher for SOC + IP than SOC + Placebo. The increase in Th1 response was significant in the SOC + IP group (p = 0.023), but not in the SOC + Placebo group (p = 0.098). The IgG antibodies reduced significantly in both the arms; however, the effect on IgM was non-significant in both the arms. COVID-19 antibodies testing was done on day 30. The antibodies were 20% more in the SOC + IP arm as compared to the SOC + Placebo; however, the effect was statistically insignificant. Due to inadequate sample size for CRP and D-Dimer analysis was not feasible; however, average 50% reduction in CRP was seen in the SOC + IP arm, whereas an increase was observed in the SOC + Placebo arm. Similar was the finding for D-Dimer. Less number of patients opted for blood withdrawal during the follow-up visits, and majority of them denied the bloodletting.

**Table 4:** Comparison of markers between and within groups.

Parameters	SOC + Placebo (n = 14)	SOC + IP (n = 15)	P-value^b^
**Total leucocyte count**			
Baseline	6257.1 ± 4488.6	7240.0 ± 2580.4	0.472
Follow-up	7200.0 ± 1669.6	7606.7 ± 1547.5	0.502
Change from baseline (95% CI)	942.6 (3005.0 to −1119.3)	366.7 (1720.2 to −986.8)	
P value^a^	0.341	0.57	
**Absolute B cell count**			
Baseline	193.4 ± 102.6	255.9 ± 154.7	0.215
Follow-up	317.9 ± 213.5	437.3 ± 270.5	0.2
Change from baseline (95% CI)	124.5 (205.1–43.8)	181.4 (282.4–80.5)	
P value^a^	0.005	0.002	
**Absolute T cell count**			
Baseline	710.5 ± 389.4	836.8 ± 455.6	0.431
Follow-up	1512.7 ± 477.8	1720.9 ± 611.7	0.319
Change from baseline (95% CI)	802.2 (1094.5–510.0)	884.1 (1230.7–537.5)	
P value^a^	<0.0001	<0.0001	
**Absolute CD3, CD4 and T helper cells**			
Baseline	399.8 ± 239.1	515.5 ± 314.8	0.278
Follow-up	834.8 ± 261.3	1055.7 ± 417.6	0.102
Change from baseline (95% CI)	435.0 (628.1–242.1)	540.2 (737.1–343.4)	
P value^a^	<0.0001	<0.0001	
**Absolute CD3: CD8**			
Baseline	270.7 ± 128.6	299.7 ± 183.5	0.629
Follow-up	571.4 ± 195.7	607.3 ± 258.8	0.679
Change from baseline	300.7 (406.1–195.4)	307.6 (455.8–159.3)	
P value^a^	<0.0001	0.001	
**Absolute T**_**reg**_**counts**			
Baseline	5.9 ± 5.0	6.5 ± 7.1	0.817
Follow-up	3.6 ± 2.7	4.8 ± 2.9	0.244
Change from baseline (95% CI)	−2.3 (0.35 to −5.1)	−1.7 (2.1 to −5.3)	
P value^a^	0.082	0.358	
**Absolute NK Cell count**			
Baseline	10.4 ± 9.0	9.9 ± 11.4	0.898
Follow-up	17.6 ± 9.5	28.3 ± 32.5	0.248
Change from baseline (95% CI)	7.2 (13.0–1.3)	18.3 (38.1 to −1.5)	
P value^a^	0.02	0.067	
**Neutrophils (%)**			
Baseline	63.7 ± 16.6	63.9 ± 17.1	0.969
Follow-up	54.4 ± 7.9	51.7 ± 9.3	0.422
Change from baseline (95% CI)	−9.3 (−1.5 to −17.0)	−12.2 (−2.6 to −21.9)	
P value^a^	0.023	0.017	
**Absolute neutrophil count**			
Baseline	4457.1 ± 4597.1	4615.4 ± 2562.2	0.914
Follow-up	3985.7 ± 1346.1	4046.1 ± 1158.0	0.902
Change from baseline (95% CI)	−3555.4 (1581.4 to −2524.3)	−569.3 (799.3 to −1937.8)	
P value^a^	0.628	0.383	
Lymphocyte (%)			
Baseline	25.7 ± 12.6	27.2 ± 14.7	0.776
Follow-up	32.8 ± 7.1	37.0 ± 7.0	0.135
Change from baseline (95% CI)	7.1 (13.7–0.5)	9.8 (18.5–1.1)	
P value^a^	0.036	0.031	
**Absolute lymphocyte counts**			
Baseline	1250.0 ± 462.7	1653.8 ± 773.1	0.109
Follow-up	2342.9 ± 734.5	2846.1 ± 810.0	0.103
Change from baseline (95% CI)	1092.9 (1515.0–670.7)	1192.3 (1698.8–685.8)	
P value^a^	<0.0001	<0.0001	
**Th cell response**			
Ab Th1			
Baseline	209.5 (45.3–419.8)	164 (27–501)	0.861
Follow-up	347 (215.8–529.3)	430 (235–683)	0.338
P value^a^	0.098	0.023	
**Ab Th2**			
Baseline	143 (66.5–230)	164 (80–398)	0.318
Follow-up	424 (343.7–593.8)	593 (351–768)	0.338
P value^a^	0.005	0.003	
**Antibody levels**			
IgG			
Baseline	1301.4 ± 284.9	1593.6 ± 309.5	0.015
Follow-up	1132.3 ± 223.9	1356.6 ± 260.5	0.021
Change from baseline (95% CI)	−169.1 (−42.9 to −295.4)	−237.0 (−79.9 to −394.1)	
P value^a^	0.012	0.007	
**IgM**			
Baseline	117.7 ± 77.2	98.8 ± 40.6	0.438
Follow-up	113.0 ± 62.9	89.9 ± 28.4	0.42
Change from baseline (95% CI)	−4.7 (8.2 to −17.5)	−8.9 (8.8 to −26.7)	
P value^a^	0.45	0.295	
**COVID Antibodies**			
	5.1 ± 2.5	6.0 ± 2.7	0.353

a Using paired t-test.

b Using t-test for independent samples.

### 3.5 Safety assessment

There were no serious adverse events in the drug arm. One SAE occurred in the SOC + Placebo arm, which was ‘prolongation of hospitalization’ due to COVID-19 related pneumonia, which got resolved later. LFT and RFT parameters were not deranged in any of the patient, confirming the safety. No drug–drug interaction with the SOC was seen.

## 4 Discussion

Since December 2019, COVID -19 pandemic has been around the globe affecting millions of individuals with significant contribution in morbidity and mortality. Despite being present for over a year, definite therapies are limited for COVID-19. Modulating immunological response with steroids has been found beneficial in hospitalized patients. However, in a large majority of patients who have mild to moderate disease, effective treatment is necessary to prevent disease progression as well as shedding of the virus to lower the transmission rates. Thus, reducing the viral load may assist in achieving this objective. Viral load independently correlates with the risk of intubation and in-hospital mortality [12,13]. We observed that SOC + IP was associated with significant reduction in viral load at day 4. Reducing the viral load can help in early symptom recovery. It was evident as NRC was significantly reduced. Reduction in NRS also suggests improvement in respiratory health. Anti-viral activity of SOC + IP therefore can be helpful in early recovery of mild to moderate COVID-19 patients. Among currently employed antiviral agents, early treatment with Favipiravir was reported to be associated with lower in VL at day 6 in hospitalized patients with COVID-19 [14].

Another antiviral agent Remdesivir was widely used. However, Remdesivir did not appear to affect rates of viral RNA load decline and mortality when compared with placebo in severe hospitalized COVID-19 patients [15]. Thus, initiating an antiviral agent before the peak viral load might be necessary. It may be difficult in clinical situation to predict the time of peak VL. In this study, SOC + IP was initiated as soon as individuals were tested positive for COVID-19, which might have helped in early improvement in clinical NRS score.

In addition to lowering VL, modulating immune response is also established strategy. In inflammatory response, Th1 pathway is associated with increase in the release of pro-inflammatory cytokines like IL-1, IL-2, IL-12, TNF-α, IFN-γ, etc. Dysregulated immune response is associated with development of cytokine storm. Most recent data demonstrated that COVID-19 might affect lymphocytes, especially T lymphocytes. The absolute number of T lymphocytes, CD4+ T cells, and CD8+ T cells decreases in the infected individuals [16]. Immunomodulatory activity of single plants as well as polyherbal formulations from Ayurveda are well reported [17]. The immunomodulation and antioxidant effect of *Zingiber officinale* extract against lead toxicity was studied by Mohamed E. et al. (2014) [18]. The phytochemical evaluation of *G. glabra* showed memory enhancement, antidepressant, antimicrobial and anticancer effect as studied by Ali Esmail [19]. Modulation of immune response was evident in our study. Though SOC + placebo showed significant effect, the change in Absolute CD3, CD4 and T helper cells was numerically higher with SOC + IP treatment. T_regs_ and their functions are compromised in severe COVID-19 patients, engendering unrestrained immune cell activation resulting in damaged lungs in severe COVID-19 patients [20]. There was less reduction of Tregs in the SOC + IP arm as compared to the SOC + placebo arm, indicating a good inflammatory control in the SOC + IP arm. Among COVID-19 patients, NK cell number and function gets reduced, resulting in the decreased clearance of infected and activated cells, and unchecked elevation of tissue-damaging inflammation markers. Restoration of NK cell effector functions has the potential to correct the delicate immune balance required to effectively overcome SARS-CoV-2 infection [21,22]. Though the statistical significance was not reached, increase in the number of NK cells was higher in SOC + IP arm than that of SOC + placebo arm. It probably indicates a potential to preserve and enhance the NK cell function that might assist in viral clearance.

The in-vitro study conducted with study drug with Remdesivir was used as a positive control for viral inhibition. Inhibition of virus replication was determined based on the fold change in the Ct value in TS-treated cells compared to the control. After 24 h, % inhibition of E-gene was 9.10% and of N gene, it was 21.93%. After 48 h, N gene inhibition was same as 24 h; however, E gene was further inhibited to 28.43% (data on shown). Thus, clinical effects of viral load reduction identified with qRT-PCR are supported by the in-vitro data.

At individual level, each ingredient has unique properties and for example, Guduchi (*T. cordifolia*), Haritaki (*T. chebula*), Amalaki (Eblica officinalis) have shown efficacy as anti-viral drugs, as an anti-oxidant, as an immune modulator. At least four natural compounds from *T. cordifolia* showed high binding efficacy against SARS-CoV-2 targets involved in attachment and replication of the virus, hence validating the merit of using *T. cordifolia* in the clinical management of infection caused by SARS-CoV-2. There is a certain efficacy of natural compounds from *T. cordifolia* against SARS-CoV-2 protease, surface glycoprotein and RNA polymerase [23,24]. Extract of *T. chebula* increased spleen lymphocyte proliferation. Based on RT-PCR analysis, the expression of cytokines (IL-2, IL-10 and TNF-α), was more in *T. chebula*-treated than in other control arms [25]. Antioxidant properties of *P. longum*, Zinziber officinale are also well-known [26,27]. Antiviral activity of Vidanga (*E. ribes*) against Influenza virus and antioxidant activity are well established [28].Anti-inflammatory, antiviral effects of Yeshtimadhu (*G. glabra*) are also well-known [29,30]. Shatavari (*A. racemosus*) has many activities including antioxidant and Immunomodulatory activities [31,32]. Minerals in the drug, Shankha (incinerated conch shell) Bhasma is helpful to tackle gastric symptoms related to the disease and Jasad Bhasma (zinc oxide) has an antiviral activity [33]. Jasad Bhasma is the purified zinc from Ayurvedic process to yield optimum effect of zinc supplementation. Due to antioxidant effects of Zinc, it protects against ROS and RNS. Zinc helps modulate cytokine release and induces proliferation of T cells and helps to maintain skin and mucosal membrane integrity. Zinc has a central role in cellular growth and differentiation of immune cells. It is essential for intracellular binding of tyrosine kinase to T cell receptors, required for T lymphocyte development and activation. Zinc supports Th1 response [34].

When we discuss about the possible mechanism of action, the approach is such that the combination acts ‘as a whole’. There are multiple phyto-ingredients and a lot of chemical entities that act on multiple targets in various systems of the body. By virtue of such entity-rich drug, a milieu is created in the body, which is prepared to take care of the infection at multiple levels, organs and systems. Since it has shown positive results in reducing viral load and immune modulation of TH1 response, the same herbo-mineral formulation can have role for prophylactic action towards the virus.

One of the limitations of the study is smaller number of participants. Currently the formulation is known as Capsule CoviLyzer™ and Capsule Abayakasthaa, and is manufactured by Ishaanav Nutraceuticals Pvt. Ltd.

With the ongoing COVID-19 pandemic, search for the potential antiviral therapies is ongoing. This polyherbal combination drug was identified to provide effective antiviral activity in mild to moderate COVID-19 patients. Potential immunomodulatory effects observed with drug can also assist in preventing inflammatory tissue damage and may help reduce the intensity of systemic inflammatory syndrome. The investigational drug was found to be safe with no serious adverse event and it can be safely given along with Standard of Care to enhance its therapeutic activity in patients with mild to moderate COVID-19 disease for reducing viral load, cytokine storm and early clinical recovery. Another study on severely affected COVID-19 patients would be worthwhile to explore of this formulation, along with the standard of care.

## Ethical approval

The study was approved by the Board of Research Studies (BORS) and Institutional Ethics Committee of Yashwantrao Chavan Memorial Hospital, Pimpri, Pune.

## Funding

The trial was funded by AMAI Trust, Pune, India.

## Author's contribution

Dr. Suresh Patankar conceptualized the study and prepared the outline & framework for conducting the same. Dr. Suresh Patankar and Dr. Anupama Gorde wrote the main manuscript text. Dr. Anupama Gorde and Dr. Tejas Shah prepared the figures. Dr. Kishor Suryawanshi, Dr. Prakash Soni with the help of Dr. Kalpana Joshi conducted the study and compiled and collated the data and results. Dr. Hrishikesh Rangnekar contributed in editing and formatting the manuscript text. Dr. Diwakar Jha shouldered the responsibility of monitoring the IP development alongwith Mr. Sagar Patankar. Mr. Sagar Patankar and Dr. Rajesh Raje managed the support staff and systems alongwith the supply of logistics.

## Data availability

Data will be made available on request.

## Declaration of competing interest

None to declare.
