# Supplementary material for: Efficacy and safety of polyherbal formulation as an add-on to standard-of-care in mild-to-moderate COVID-19: A randomized, double-blind, placebo-controlled trial
Source: J Ayurveda Integr Med. 2022 Oct 24;13(4):100653. doi: 10.1016/j.jaim.2022.100653 (PMC9595378; doi:10.1016/j.jaim.2022.100653)
Supplement: Multimedia component 1 [file mmc1.docx]

**REFERENCES ON THE INVESTIGATIONAL PRODUCTS (IP 1 & 2)**

**IP 1 CONTENTS**

1. Ginger extract (Rhizome) Zingiber officinale - 50mg

2. Vidanga extract (fruit) Embelia ribes- 100mg

3. Yeshtimadhu (root) Glycyrrhiza glabra- 55mg

4. Jasad bhasm(oxide of zinc)- 20 mg

5. Shankh bhasm (calx of conch shell) -125 mg

**1**. **Ginger extract (Rhizome) Zingiber officinale**

a) We found that DZO inhibits LPS-induced inflammation via regulation of NF-κB and MAP kinases. DZO significantly inhibits the production of IFN-γ and IL-6 and suppresses NF-κB by degradation of IκB-α. These activities appear to be mediated via downregulation of the ERK1/2, SAPK/JNK, and p38 MAP kinases signaling pathways and suppression of iNOS and COX-2. Our data provide evidence for a mechanism by which DZO acts as an anti-inflammatory agent. Strategic use of DZO in treating inflammatory diseases could provide therapeutic benefits for future clinical use. (https://www.ncbi.nlm.nih.gov/pmc/articles/PMC3712229)

# b) Ginger is one of the most effective natural immunomodulator. It is found that ginger inhibited lymphocyte proliferation; this was mediated by reductions in IL-2 and IL-10 production.

# Reference: Wilasrusmee C, Siddiqui J, Bruch D, Wilasrusmee S, Kittur S, Kittur DS. In vitro immunomodulatory effects of herbal products. American Surgeon. 2002; 68:860-864

# c) Aqueous ginger extract significantly increased the production of IL-1β, IL-6 and TNF-α in activated peritoneal mouse macrophages and splenocyte proliferation and cytokine production.

# Reference: Ryu HS, Kim HS. Effect of Zingiber officinale Roscoe extracts on mice immune cell activation. Korean Journal of Nutrition. 2004; 37(1):23-30.

# d) Ginger rhizome diet for 12 weeks showed increased haematocrit, haemoglobin, erythrocyte, MCH, MCHC, WBC values and neutrophils percentage. Ginger essential oil showed improvement in humoral and cell mediated immune response in immune suppressed mice.

# Reference: Carrasco FR, Schmidt G, Romero AL, Sartoretto JL, Caparroz-Assef SM, Bersani-Amado CA “et.al.” Immunomodulatory activity of Zingiber officinale Roscoe, Salvia officinalis L. and Syzygium aromaticum L. essential oils: evidence for humor- and cell-mediated responses. Journal of Pharmacy and Pharmacology. 2009; 1(7):961-967.

# e) The powdered ginger rhizome is capable to improve non-specific immune response.

# Reference: Haghighi M, Rohani MS. The effects of powdered ginger (Zingiber officinale) on the haematological and immunological parameters of rainbow trout Oncorhynchus mykiss. Journal of medicinal Plant and Herbal therapy research. 2013; 1:8-12

# 2. Embelia Ribes (Vidanga extract)

# a) Antioxidant activity

# Reference: Joshi R,Kumar J P,Mukherjee T.Free radical scanvanging reaction and antioxidant activity of Embelin:Biochemical and Pulse radiolytic studies.Chem Biol interact.2007.167(2):125-134 (Rat)

# b) Neuroprotective activity

# Reference: Thippeswamy B S etal,Protective effect of EmbelinFrom Embelia Ribes Burm,against transitent global ischemiainduced brail damage in rats.Undam clin Pharmacol.2008.22(3):305-314

# c) Cardioprotective activity

# Reference: U bhandari et al Cardioprotective effect of aqueous extract of Embelia ribes Burm fruits against isoportenol induced myocardial infarction in albino rats.Indian Journal of Experimental Biology 2008.46:35-40

# Reference: Radhakrishnan N etal, Inhibition of UVB induced oxidative damage and apoptotic biochemical changes in human lymphocytes by 2,5-dihydroxy-3undecyl-1,4-benzoquinone(embelin).Int.J.Radiat Biol.2012.88(8):575-82(peripheral blood human lymphocytes)

**e) Embelia ribes extract has shown to enhance antioxidant defense and neuroprotective activity**

**Reference:** Asian Jr. Multidisciplinary Studies, 2015, Vol.3 (2)

f) Our findings indicate the utility of embelin as an adjuvant for oncolytic viro-immunotherapy.

**Reference:** OncoTargets and Therapy, 17 Feb, 2020, Vol. 13, 1421-1429

# 3. Glycyrrhiza Glabra (Yeshtimadhu )

### a) Antioxidant activity

The antioxidant activity of G. glabra is one of the major reasons for its uses. The phenolic content is probably responsible for the powerful antioxidant activity observed (Rackova et al., [2007](https://www.ncbi.nlm.nih.gov/pmc/articles/PMC7167772/#ptr6178-bib-0096)). Varsha and Sonam ([2013](https://www.ncbi.nlm.nih.gov/pmc/articles/PMC7167772/#ptr6178-bib-0129)) attributed this activity to flavonoids, whereas Singh et al. ([2015](https://www.ncbi.nlm.nih.gov/pmc/articles/PMC7167772/#ptr6178-bib-0114)) reported that mostly isoflavones, such as glabridin, hispaglabridin A, and 30‐hydroxy‐4‐O‐methylglabridin, are the responsible compounds. Biondi, Rocco, and Ruberto ([2003](https://www.ncbi.nlm.nih.gov/pmc/articles/PMC7167772/#ptr6178-bib-0011)) reported a huge antioxidant activity of the dihydrostilbene derivates present in G. glabra leaves. Also, licochalcones B and D are present in G. glabra, showing a strong scavenging activity on DPPH radical and the ability to inhibit the microsomal lipid peroxidation (Biondi et al., [2003](https://www.ncbi.nlm.nih.gov/pmc/articles/PMC7167772/#ptr6178-bib-0011); V. Sharma, Katiyar, & Agrawal, [2016](https://www.ncbi.nlm.nih.gov/pmc/articles/PMC7167772/#ptr6178-bib-0111)).

These phenolic compounds are effective in the protection of biological systems against oxidative stress, being able to inhibit the onset of skin damages (Haraguchi et al., [1998](https://www.ncbi.nlm.nih.gov/pmc/articles/PMC7167772/#ptr6178-bib-0045))..

### b)Anti‐inflammatory activity

The anti‐inflammatory activity of G. glabra and its use in the treatment of inflammatory diseases have been documented since ancient times (R. Yang, Yuan, Ma, Zhou, & Liu, [2017](https://www.ncbi.nlm.nih.gov/pmc/articles/PMC7167772/#ptr6178-bib-0140)). Shalaby, Ibrahim, Mahmoud, and Mahmoud ([2004](https://www.ncbi.nlm.nih.gov/pmc/articles/PMC7167772/#ptr6178-bib-0107)) evaluated the anti‐inflammatory activity of G. glabra in male rats after 4 weeks of food intake. The authors observed a significant decrease in the total cholesterol and triglyceride levels as well as in the levels of serum liver enzymes. Harwansh, Patra, Pareta, Singh, and Biswas ([2011](https://www.ncbi.nlm.nih.gov/pmc/articles/PMC7167772/#ptr6178-bib-0046)) reviewed the positive effects of G. glabra on the treatment of the upper respiratory tract and gastric system diseases. These pharmacological effects were due to an increase in the secretion of serotonin and prostaglandins in the stomach that led to a decrease of gastric inflammation (Bahmani et al., [2014](https://www.ncbi.nlm.nih.gov/pmc/articles/PMC7167772/#ptr6178-bib-0008)). Different authors described that the anti‐inflammatory action is primary mediated by glycyrrhizin, which in vitro could inhibit factors responsible for inflammation as well as promote the healing of stomach and mouth ulcers (Rackova et al., [2007](https://www.ncbi.nlm.nih.gov/pmc/articles/PMC7167772/#ptr6178-bib-0096); Yin et al., [2017](https://www.ncbi.nlm.nih.gov/pmc/articles/PMC7167772/#ptr6178-bib-0143)). In fact, the anti‐inflammatory effects of glycyrrhizin were described as similar to those of glucocorticoids and mineralocorticoids (Kageyama, Suzuki, & Saruta, [1994](https://www.ncbi.nlm.nih.gov/pmc/articles/PMC7167772/#ptr6178-bib-0064)). Furthermore, G. glabra is used in renal and liver complications on the basis of its strong anti‐inflammatory effects (Y. Xiao et al., [2010](https://www.ncbi.nlm.nih.gov/pmc/articles/PMC7167772/#ptr6178-bib-0138)). Y. Xiao et al. ([2010](https://www.ncbi.nlm.nih.gov/pmc/articles/PMC7167772/#ptr6178-bib-0138)) reported the inhibition of liver granuloma formation and the inflammatory cytokine production by glycyrrhizin, whereas X. R. Wang, Hao, and Chu ([2017](https://www.ncbi.nlm.nih.gov/pmc/articles/PMC7167772/#ptr6178-bib-0135)) described the anti‐inflammatory effects on endometriosis. Moreover, Liu et al. ([2017](https://www.ncbi.nlm.nih.gov/pmc/articles/PMC7167772/#ptr6178-bib-0077)) proved the anti‐inflammatory activity of glabridin on RAW cells

### c) Antiviral activity

The antiviral activity of G. glabra extracts against different viruses has been reported, including herpes simplex, Varicella zoster, Japanese encephalitis, influenza, and vesicular stomatitis virus (L. Wang, Yang, et al., [2015](https://www.ncbi.nlm.nih.gov/pmc/articles/PMC7167772/#ptr6178-bib-0131)). Different studies have demonstrated that two triterpenoids are responsible for the antiviral activity reported: glycyrrhizin and 18β‐glycyrrhetinic acid (L. Wang, Yang, et al., [2015](https://www.ncbi.nlm.nih.gov/pmc/articles/PMC7167772/#ptr6178-bib-0131)). These compounds have the ability to inhibit virus gene expression and replication, decreasing the adhesion force and stress and reducing HMGB1 binding to DNA (L. Wang, Yang, et al., [2015](https://www.ncbi.nlm.nih.gov/pmc/articles/PMC7167772/#ptr6178-bib-0131)). Also, they can enhance host cell activities by blocking the degradation of IκB enzyme involved in the propagation of the cellular response to inflammation, activating T lymphocyte proliferation, and suppressing host cell apoptosis (L. Wang, Yang, et al., [2015](https://www.ncbi.nlm.nih.gov/pmc/articles/PMC7167772/#ptr6178-bib-0131)). The antiviral mechanisms of both compounds are similar, inhibiting the adsorption and penetration of the virus in the early steps of the replicative cycle. Nevertheless, Cinatl et al. ([2003](https://www.ncbi.nlm.nih.gov/pmc/articles/PMC7167772/#ptr6178-bib-0025)) reported that these active principles are less effective if added during the adsorption period than after virus adsorption. On the other hand, Soufy et al. ([2012](https://www.ncbi.nlm.nih.gov/pmc/articles/PMC7167772/#ptr6178-bib-0118)) found that glycyrrhizin has excellent immunostimulant properties and induces a synergistic effect with duck hepatitis virus (DHV) vaccine by activating T lymphocyte proliferation. Thus, the treatment with glycyrrhizin alone or in combination with DHV vaccine could lead to an immune stimulation and antiviral effect against DHV (Soufy et al., [2012](https://www.ncbi.nlm.nih.gov/pmc/articles/PMC7167772/#ptr6178-bib-0118)).

Herpes simplex virus (HSV) is one of the most common viruses infecting humans and animals. During HSV infection, the cellular adhesion is increased, playing a key role in inflammatory response. W. Huang et al. ([2012](https://www.ncbi.nlm.nih.gov/pmc/articles/PMC7167772/#ptr6178-bib-0056)) reported that the adhesion force and stress between the cerebral capillary vessel endothelial cells and the polymorphic nuclear leukocytes were amplified during HSV infection. Glycyrrhizin stimulates the mouse defence system against HSV‐1 infection (Sekizawa, Yanagi, & Itoyama, [2001](https://www.ncbi.nlm.nih.gov/pmc/articles/PMC7167772/#ptr6178-bib-0106)). Furthermore, glycyrrhizic acid was found to have a distinctive effect against Kaposi sarcoma‐associated herpes virus (KSHV). It was proved that glycyrrhizic acid could terminate the latent infection of KSHV when all current drugs are ineffective (Damle, [2014](https://www.ncbi.nlm.nih.gov/pmc/articles/PMC7167772/#ptr6178-bib-0026)). Also, glycyrrhizic acid down‐regulates the expression of latency‐associated nuclear antigen in B lymphocytes leading to natural cell death (apoptosis) of the KSHV‐infected cells (Damle, [2014](https://www.ncbi.nlm.nih.gov/pmc/articles/PMC7167772/#ptr6178-bib-0026)). Recently, the antiviral activity of glycyrrhizin against severe acute respiratory syndrome virus was evaluated (Cinatl et al., [2003](https://www.ncbi.nlm.nih.gov/pmc/articles/PMC7167772/#ptr6178-bib-0025)). Glycyrrhizin affects the cellular signalling pathways such as protein kinase C, casein kinase II, and transcription factors, namely, activator protein 1 and nuclear factor κB (Cinatl et al., [2003](https://www.ncbi.nlm.nih.gov/pmc/articles/PMC7167772/#ptr6178-bib-0025)). Furthermore, glycyrrhizin and its aglycone, 18β‐glycyrrhetinic acid, up‐regulate the expression of inducible nitric oxide synthase and the production of nitric oxide in macrophages (Cinatl et al., [2003](https://www.ncbi.nlm.nih.gov/pmc/articles/PMC7167772/#ptr6178-bib-0025)). Zhang et al. also reported that glycyrrhizin reduces the expression of proinflammatory cytokines affecting coxsackievirus B3‐induced myocarditis (L. Wang, Yang, et al., [2015](https://www.ncbi.nlm.nih.gov/pmc/articles/PMC7167772/#ptr6178-bib-0131); Zhang, Song, & Zhang, [2012](https://www.ncbi.nlm.nih.gov/pmc/articles/PMC7167772/#ptr6178-bib-0149)). Also, the activity against human immunodeficiency virus (HIV) was evaluated (Sasaki et al., [2002](https://www.ncbi.nlm.nih.gov/pmc/articles/PMC7167772/#ptr6178-bib-0102)). Glycyrrhizin has been used to treat patients with HIV‐1 (L. Wang, Yang, et al., [2015](https://www.ncbi.nlm.nih.gov/pmc/articles/PMC7167772/#ptr6178-bib-0131)). The results revealed a low concentration of P24 antigen in patients, probably due to the up‐regulation of chemokines (Sabde et al., [2011](https://www.ncbi.nlm.nih.gov/pmc/articles/PMC7167772/#ptr6178-bib-0100)).

Intravenous glycyrrhizin has been employed for more than 20 years in Japan for the treatment of chronic hepatitis (van Rossum et al., [1999](https://www.ncbi.nlm.nih.gov/pmc/articles/PMC7167772/#ptr6178-bib-0128)). Glycyrrhizin, when compared with the placebo, presents clinical interest for the possible treatment of chronic hepatitis C, inducing a significant reduction of the serum aminotransferases and an improvement in the liver histology (Ploeger et al., [2001](https://www.ncbi.nlm.nih.gov/pmc/articles/PMC7167772/#ptr6178-bib-0094)). Also, it prevents the development of hepatocellular carcinoma in chronic hepatitis C (van Rossum, Vulto, de Man, Brouwer, & Schalm, [1998](https://www.ncbi.nlm.nih.gov/pmc/articles/PMC7167772/#ptr6178-bib-0127)). Intravenous glycyrrhizin can be also used for the treatment of acute‐onset autoimmune hepatitis (Yasui et al., [2011](https://www.ncbi.nlm.nih.gov/pmc/articles/PMC7167772/#ptr6178-bib-0142)). Another study shows that glycyrrhizin interferes with highly pathogenic H5N1 influenza A virus replication (Michaelis et al., [2011](https://www.ncbi.nlm.nih.gov/pmc/articles/PMC7167772/#ptr6178-bib-0081)).

<https://www.ncbi.nlm.nih.gov/pmc/articles/PMC7167772/>

d) The activity of licorice on the immune system has been described as "nonspecific" by most investigators. This means licorice stimulates, activates or promotes an immune response in multiple ways.

**Reference:** Chavali SR, et al. An in vitro study of immunomodulatory effects of some saponins. Intl J Immunopharmacol 1987;9:675.

e) Licorice also appears to stimulate the production of gamma-interferon by lymphocytes.

**Reference:** Nara IZ. The role of interferon-gamma (IFN-gamma) producing cells in clinical immunology. Chem Abstracts 1984;35:424.

# *4.* Shankha Bhasma *-*it is ayurvedic preparation of Calcium prepared from conch shells.

The XRD pattern of both raw and finished conch ([Figure 2](https://www.ncbi.nlm.nih.gov/pmc/articles/PMC6266202/figure/marinedrugs-16-00450-f002/)) when simulated with standards, clearly indicated that raw conch is aragonite in nature, while after incineration this aragonite structure got rearranged to calcite form. SEM analysis of raw conch and finished product also confirmed the structural transformation of aragonite to calciteCalcium carbonate nanoparticles have wide range of applications in drug delivery system due to its unique properties like accessibility, low cost, safety, biocompatibility, pH sensitive properties and slow biodegradability [[42](https://www.ncbi.nlm.nih.gov/pmc/articles/PMC6266202/#B42-marinedrugs-16-00450)].

All these experimental studies conducted through advanced instruments are helpful to understand the pharmaceutical transformation of aragonite conch into better absorbed calcite known as Shankha Bhasma in Ayurveda. Shankha Bhasma being a traditional drug, the authors is clinically investigating the role of Shankha Bhasma as adjunct treatment in GI malignancies, mainly stomach and colo-rectal cancers, by assessing the GI symptoms like nausea, vomiting, anorexia, flatulence, indigestion, mucositis, hyperacidity caused due to these malignancies as well as toxicities of chemotherapy.

<https://www.ncbi.nlm.nih.gov/pmc/articles/PMC6266202/>

**5. Jasad bhasma**

- Calcined Zinc is the purified zinc from Ayurvedic process to yield optimum effect of zinc supplementation.
- Due to antioxidant effects of Zinc it protects against ROS and RNS. Zinc helps modulate cytokine release and induces proliferation of T cells and helps to maintain skin and mucosal membrane integrity.
- Zinc has a central role in cellular growth and differentiation of immune cells. It is essential for intracellular binding of tyrosine kinase to T cell receptors, required for T lymphocyte development and activation. Zinc supports Th1 response.

**Reference:** Nutrients, 2018 Oct; 10(10): 1531.

- Zinc deficiency has been noted to result in increased susceptibility to infectious disease. Suboptimal Zinc status has also been associated with decreased T-cell function and antibody responses. If the Zinc deficiency is corrected, immune status is restored. Zinc is an essential cofactor for thymulin, a peptide hormone that plays a key role in T-cell maturation

**Reference:** www.nap.edu/read/6450/chapter/23

Yashada Bhasma acts as a cytostatic drug in human Pancreatic ductal adenocarcinoma, due to their ability to induce cell growth arrest. This may open up new possibilities for cancer control. The current findings admit new assurance for the treatment of pancreatic cancers by developing a unique and highly effective therapeutic agent.

**Reference:** https://www.ncbi.nlm.nih.gov/pmc/articles/PMC6891994/

**IP2 CONTENTS**

1.Haritaki- Terminalia chebula (fruit)

2.Giloy extract- Tinospora cordifolia (whole plant)

3.Shatavari- Asparagus recemosus (root)

4.Aamalaki - Emblica officinalis (fruit)

5. Ashwagandha- Withania somnifera (stem)

6.Pippali- Piper longum (fruit)

7. Jasad Bhasma

# 1. Emblica Officinalis (Aamalaki)

a) AFE showed significant gastric acid resistance and was also found to be thermostable against wet heat. Excellent α‐amylase, α‐glucosidase, and DPP‐4 inhibitory activities of AFE, as well as antioxidant activities, strongly recommend its use for the management of type 2 diabetes mellitus. © 2019 The Authors. Journal of The Science of Food and Agriculture published by John Wiley & Sons Ltd on behalf of Society of Chemical Industry.

b) Apart from various properties, immunomodulation is one of the prominent property of this plant. E. officinalis has shown to modulate immune system functions.

**Reference:** Int. J. Curr. Microbiol. App. Sci. 2017, Vol. 6(7), 4267-4280

c) E. officinalis-treated mice as compared to control group indicating the role of extract in macrophage activation. This was accompanied by burst of oxidative metabolism generating ROS detected through NBT assay, confirmed the intracellular killing property of phagocytosing macrophages.

**Reference:** Suja RS, Nair AM, Sujith S, Preethy J, Deepa AK. Evaluation of immunomodulatory potential of Emblica officinalis fruit pulp extract in mice. Indian J Anim Res. 2009;113:103–6.

# 2.Terminalia Chebula (Haritaki)

A0The results from various free radical scavenging systems revealed that all the fruit extracts were individually strong antioxidants, with some varying scavenging activities for different ROS at different magnitudes of potency. Furthermore, evaluation of in vivo antioxidant activity of these fruit extracts has also provided interesting results that might be beneficial for the pharmacological use of these plants in clinical trials.. Further, the isolation of the compounds responsible for the antioxidant activity has to be taken up which may result in modern drugs from these plants.

**Reference:** <https://www.ncbi.nlm.nih.gov/pmc/articles/PMC2887379/>

b) Terminalia chebula extract displays immunostimulatory effect in relation to antigenic s. Terminalia chebula extract produced a dose-dependent increase in both the parameters (i.e., antibody production and delayed-type hypersensitivity). Therefore, is concluded that the aqueous extract of fruit of Terminalia chebula has promising immunostimulant properties. The biologically active compounds such as chebulagic acid, gallic acid and ellagic acid make T. chebula highly potent antioxidant, which may be responsible for its immunomodulatory activity.

**Reference:** http://doi.org/10.1080/13880200500530542

c) Its extract neutralizes reactive oxygen species (ROS) and scavenges free radicals. The free radicals are responsible for causing inflammation by stimulating release of cytokines such as IL-1, TNF-α and IFN-β, which stimulate additional neutrophils and macrophages at site of inflammation. Thus, different antioxidants of the extract exhibit immunosuppressive properties, which help in neutralizing these important inflammatory mediators.

# 3.Tinospora Cordifolia (Giloy extract)

#### A)Anticomplement activity and immunomodulating activity

Kapil et al., Studied the syringin (TC-4) and cordiol (TC-7) isolate from T. cordifolia inhibited the in-vitro immune hemolysis of antibody-coated sheep erythrocytes by guinea pig serum. Immune hemolysis was reduced due to inhibition of the C3-convertase of the classical complement pathway. The compounds of T. cordifolia rise to significant increases in IgG antibodies in guinea pig serum. Cordioside (TC-2), cordiofolioside A (TC-5) and cordiol (TC-7) activated macrophase with increasing incubation times [[96]](https://www.ncbi.nlm.nih.gov/pmc/articles/PMC6827274/#bib96). Sharma et al., isolated and characterised different classes of active compounds reported their mmunomodulatory activity [[7]](https://www.ncbi.nlm.nih.gov/pmc/articles/PMC6827274/#bib7).

**Reference:** <https://www.ncbi.nlm.nih.gov/pmc/articles/PMC6827274/>

b) Tinospora cordifolia is an important medicinal plant. Through centuries, it has been extensively used in traditional medicinal reparations for treating various ailments. A variety of plant-derived materials polysaccharides, lectins, peptides etc. have been reported to stimulate immune system. Extract of T. cordifolia posses good immunomodulatory activity

**Reference:** Int.Jr. Cur. Pharm. Res., 2010, Vol.2(4), 52-54

# 4. Withania Somnifera (*Ashwagandha )*

a. Anti-oxidant and hepatoprotective activities

Lipid peroxidation activity of aqueous suspension of roots was investigated by administration to mice and rabbits at a dose of 100 mg/kg after 6 hr intervals. The concentration of lipid peroxide was decreased in K. pneumoniae and S. aureus which advocated the prophylactic activity against stress induce lipid peroxidation ([126](https://www.ncbi.nlm.nih.gov/pmc/articles/PMC7811807/#B126)). It was suggested that the anti-oxidant potential of withanolides might be due to the hydroxylated long chain of the carbon-bearing acyl group. Other compounds such as sitoindosides VII-X and withaferin A were investigated as potent initiators for free-radical scavenging enzymes, catalase, glutathione peroxidase, and superoxide dismutase in the striatum and frontal cortex of rat’s brain ([160](https://www.ncbi.nlm.nih.gov/pmc/articles/PMC7811807/#B160)).

Another study revealed the protective effect of aqueous extract of the whole plant (500–1000 mg/kg of body weight) in paracetamol-induced hepatotoxicity. The extract reversed the effects of hepatotoxicity by lowering the concentration of liver marker enzyme, bilirubin, with improvement in protein contents ([161](https://www.ncbi.nlm.nih.gov/pmc/articles/PMC7811807/#B161)). Alkaloids (withanamides A-I) extracted and purified from W. somnifera were assessed for anti-oxidant activity using a large unilamellar vesicle model. It was disclosed that withanamides (A-I) isolated from the plant fruits retarded lipid peroxidation significantly at a concentration of 0.5-1 μg/ml. It was also noticed that withanoside V displayed prominent free radical scavenging activity at 10 μg/ml concentration ([98](https://www.ncbi.nlm.nih.gov/pmc/articles/PMC7811807/#B98), [201](https://www.ncbi.nlm.nih.gov/pmc/articles/PMC7811807/#B201)). Elevation in the enzymes showed increased anti-oxidant potential with a protective effect on neural tissues ([202](https://www.ncbi.nlm.nih.gov/pmc/articles/PMC7811807/#B202)-[205](https://www.ncbi.nlm.nih.gov/pmc/articles/PMC7811807/#B205)).

Aqueous extract of the roots was tested for the anti-oxidant effect in male albino rats against cypermethrin induced oxidation. Extract, when administered at a dose of 5 ml (10% root’s extract) for 60 days to male albino rats, showed the complete restoration of all biochemical and hematological parameters ([206](https://www.ncbi.nlm.nih.gov/pmc/articles/PMC7811807/#B206)). Co-administration of methanolic extract of roots of W. somnifera, leaves of Ocimum sanctum, and rhizome of Zingiber officinale reduced tenuous physical exercise and swimming-induced oxidative damage in Wistar rats. The stresses significantly elevated the number of free radicals which lowered the activity of catalase, superoxide dismutase, and glutathione-S-transferase in secondary sex organs. Co-administration of the aforesaid extracts at a dose of 0.5 ml/100 g of body weight helped to increase anti-oxidant activity with regaining a reasonable level of enzymes ([55](https://www.ncbi.nlm.nih.gov/pmc/articles/PMC7811807/#B55),[207](https://www.ncbi.nlm.nih.gov/pmc/articles/PMC7811807/#B207)).

Glycowithanolides (sitoindosides VII-X, withaferin A) isolated and purified from roots of W. somnifera were administered to rats at doses of 10, 20, and 50 mg/kg of body weight for 10 days. The extracts reduced iron-induced hepatotoxicity due to the anti-oxidant activity of glycowithanolides ([208](https://www.ncbi.nlm.nih.gov/pmc/articles/PMC7811807/#B208)). The powder of roots affected the circulatory level of urea, ammonia, lipid peroxidation products (hydroperoxides, thiobarbituric acid reactive substances), and liver marker enzymes (alanine transaminase, aspartate transaminase, and alkaline phosphatase) showing hepatoprotective potential.  The plant elevated the level of hepatic protection by affecting the concentration of liver markers and lipid peroxidation products in experimental hyperammonemia. The hepatoprotective activity might be mediated by the controlling mechanism of alkaloids, withanolides, flavonoids, urea, and urea related compounds ([209](https://www.ncbi.nlm.nih.gov/pmc/articles/PMC7811807/#B209)). Lesions induced by carbendazim in the liver and kidney were completely cured using the powder of plant roots for 48 days ([210](https://www.ncbi.nlm.nih.gov/pmc/articles/PMC7811807/#B210)). Methanolic extract of the plant exhibited significant free radical scavenging potential and protected DNA damage induced by hydrogen peroxide ([211](https://www.ncbi.nlm.nih.gov/pmc/articles/PMC7811807/#B211)).

**b. Immunomodulatory activity and hematopoiesis**

An increase in the production of nitric oxide owing to activation of nitric oxide synthase in mouse macrophages was observed after the administration of methanolic extract of the plant roots (1–256 μg/ml) ([94](https://www.ncbi.nlm.nih.gov/pmc/articles/PMC7811807/#B94)). Evaluation of the immunomodulatory effect of purified sitoindoside IX and X from the plant on the central nervous system as anti-stress agents was studied at a dose rate of 100–400 g/mouse. It was concluded that significant activation and mobilization of peritoneal macrophages and phagocytosis enhanced the lysosomal enzymes secreted by the macrophages. It was further confirmed that sitoindosides reduced the deficits in the cerebral function of the geriatric population ([108](https://www.ncbi.nlm.nih.gov/pmc/articles/PMC7811807/#B108)). In vitro and in vivo immunomodulatory effect of plant root powder was evaluated and the potent inhibitory effect on mitogen-induced lymphocyte proliferation with delayed hypersensitivity in mice was explored ([174](https://www.ncbi.nlm.nih.gov/pmc/articles/PMC7811807/#B174)). Immunomodulatory effects of alcoholic extract of the plant roots were assessed in cyclophosphamide, azathioprine, or prednisolone myelosuppression models in mice. The extract enhanced the number of blood cells, bone marrow cellularity, and α-esterase positive cell number count ([127](https://www.ncbi.nlm.nih.gov/pmc/articles/PMC7811807/#B127), [211](https://www.ncbi.nlm.nih.gov/pmc/articles/PMC7811807/#B211), [212](https://www.ncbi.nlm.nih.gov/pmc/articles/PMC7811807/#B212)).

It was discovered recently that the mechanism of immunomodulation involved phytochemicals such as 2,3-dihydrowithaferin -A-3-β-O-sulfate, daucosterol, withasomniferol-A, withaferin-A, and β-sitosterol, which regulated multiple immunity pathways via bioactive-targets and protein-protein interactions ([213](https://www.ncbi.nlm.nih.gov/pmc/articles/PMC7811807/#B213)). In a similar study, it was found that a mixture of sitoindosides IX, X, glycol, and anolides isolated from the plant statistically enhanced the immunomodulatory effect by activation of macrophages and lysosomal enzymes ([214](https://www.ncbi.nlm.nih.gov/pmc/articles/PMC7811807/#B214)). The aqueous whole plant extract, when administered to albino rats, showed a significant increase in the production of antibodies which reduced mortality with improved immune response ([215](https://www.ncbi.nlm.nih.gov/pmc/articles/PMC7811807/#B215)).

**Reference:** <https://www.ncbi.nlm.nih.gov/pmc/articles/PMC4027291/>

c) W. somnifera is report to have immunostimulatory activity: Treatment with Ashwagandha was accompanied by significant increases in hemolytic antibody responses towards human erythrocytes.

**Reference:** J.Ethnopharmacol,. 1996, Feb; 50(2), 69-72

d) Administration of WS also showed an enhancement in phagocytic activity of peritoneal macrophages when compared to control in mice. These results confirm the immunomodulatory activity of WS extract in indigenous medicine

**Reference:** L. Davis, G. Kuttan. Immunomodulatory activity of Withania somnifera. J. Ethnopharmacol. 71(1-2): 193-200 (2000).

e) The role of WS as immunomodulator has been extensively studied. In a mouse study, WS root extract enhanced total white blood cell count. In addition, this extract inhibited delayed-type hypersensitivity reactions and enhanced phagocytic activity of macrophages when compared to a control group.

**Reference:** L. Davis, G. Kuttan. Effect of Withania somnifera on cell mediated immune responses in mice. J. Exp. Clin. Cancer Res. 21(4): 585-590 (2002)

# 5. Asparagus Racemosus (Shatavari)

### a. Immunomodulatory activity

Immunomodulating property of A. racemosus has been shown to protect the rat and mice against experimental induced abdominal sepsis[[65]](https://www.ncbi.nlm.nih.gov/pmc/articles/PMC4027291/#b65),[[66]](https://www.ncbi.nlm.nih.gov/pmc/articles/PMC4027291/#b66). Oral administration of decotion of powdered root of A. racemosus been reported to produce leucocytosis and predominant neutrophilia along with enhanced phagocytic activity of the macrophages and polymorphs. Parcentage mortality of A. racemosus treated animals was founded significantly reduced while survival rate was comparable to that of the group treated with a combination of metronidazole and gentamicin. Since A. racemosus is reported to be devoid of antibacterial action, so protection offered by A. racemosus against sepsis by altering function of macrophages, indicating its possible immunomodulatory property[[65]](https://www.ncbi.nlm.nih.gov/pmc/articles/PMC4027291/#b65),[[66]](https://www.ncbi.nlm.nih.gov/pmc/articles/PMC4027291/#b66).

### b. Immunoadjuvant potential activity

The immunoadjuvant potential of A. racemosus aqueous extract root extract was evaluated in experimental animals immunized with diphtheria, tetanus, pertussis vaccine. Immunostimulation was evaluated using serological and hematological parameters. Oral administration of test material at 100 mg/kg per day dose for 15 d resulted significant increase in antibody titre to Boredtella pertussis as compared to untreated (control) animals. Results indicate that the treated animals did show significant increase in antibody titre as compared to untreated animals after change. Applications of test material as potential immunoadjuvant bring less morbidity and mortality to experimental animals[[67]](https://www.ncbi.nlm.nih.gov/pmc/articles/PMC4027291/#b67)–[[69]](https://www.ncbi.nlm.nih.gov/pmc/articles/PMC4027291/#b69).

**Reference:** <https://www.ncbi.nlm.nih.gov/pmc/articles/PMC4027291/>

c) Tinospora cordifolia is an important medicinal plant. Through centuries, it has been extensively used in traditional medicinal reparations for treating various ailments. A variety of plant-derived materials polysaccharides, lectins, peptides etc. have been reported to stimulate immune system. Extract of T. cordifolia posses good immunomodulatory activity

**Reference:** Int.Jr. Cur. Pharm. Res., 2010, Vol.2(4), 52-54

# 6. Piper Longum-(Pippali)

a) In conclusion, this study has undoubtedly provided scientific confirmation and evidence for the safe use of the roots of Piper longum by traditional healers in the treatment of diabetes. However the nature of the active principle(s) responsible for all these positive effects requires further investigation.

**Reference:** <https://www.ncbi.nlm.nih.gov/pmc/articles/PMC3583796/>

b) Piper longum is well known for its medicinal and pharmaceutical importance.The specific and nonspecific immune-stimulatory actions of P. longum fruits have been evaluated by hemagglutination titer, macrophage migration index, and phagocytic index. It is found to activate macrophages, as shown by an increased macrophage migration index and phagocytic index, indicating immune-stimulatory activity.

**Reference:** Tripathi DM, N Gupta, V Lakshmi, KC Saxena and AK Agrawal, 1999. Antigiardial and immunostimulatory effect of Piper longum on giardiasis due to Giardia lamblia. Phytother. Res., 13: 561-565.

c) Tests such as haemagglutination titre (HA), macrophage migration index (MMI) and phagocytic index (PI) have demonstrated the immunostimulatory action of P. longum to be both specific and non specific.

**Reference:** Int. Jr. Res. Ayur & Pharm. 2011 Vol 2(1). 157-161.
