# Supplementary material for: Efficacy and safety of polyherbal formulation as an add-on to standard-of-care in mild-to-moderate COVID-19: A randomized, double-blind, placebo-controlled trial
Source: J Ayurveda Integr Med. 2022 Oct 24;13(4):100653. doi: 10.1016/j.jaim.2022.100653 (PMC9595378; doi:10.1016/j.jaim.2022.100653)
Supplement: Multimedia component 2 [file mmc2.docx]

**Table 1S**: Scientific description of investigational products

| S. No. | Ingredients | Botanical/Scientific name | Family name | Parts | Quantity | Standardization | Method of analysis |
| --- | --- | --- | --- | --- | --- | --- | --- |
|  |  |  |  |  |  |  |  |
| **IP1** | | | | | | | |
| 1 | Vidanga Extract | Embelia ribes | Primulaceae | Seed | 125mg | Embelin | HPLC |
| 2 | Ginger Extract | Zingiber officinalis | Zingiberaceae |  |  | Gingerols | HPLC |
| 3 | Yeshtimadhu | Glycyrrhiza glabra | Fabaceae | Rhizome | 80mg | Glycyrrhizin | Gravimetric |
| 4 | Shankha bhasma | Calx of Conch shel | NA | Powder | 125mg | Calcium | Atomic Absorption Spectrophotometry |
| 5 | Jasad bhasma | Zinc | NA | Powder | 20mg | Zinc | UV Visible Spectrophotometry |
| **IP2** | | | | | | | |
| 1 | Haritaki | Terminalia chebula | Combretaceae | Root | 122 mg | Chebulinic acid | HPLC |
| 2 | Giloy Extract | Tinospora cordifolia | Menispermaceae | Rhizome | 95mg | Cordiofolioside A | HPLC |
| 3 | Shatavari | Asparagus | Asparagaceae | Root | 60mg | Shatawarin IV | HPLC |
| 4 | Aamalaki | Emblica officinalis | Phyllanthaceae | Fruit | 85mg | Gallic and Ellagic Acid | HPLC |
| 5 | Pippali | Piper longum | Piperaceae | Fruit | 2mg | Piperine | HPLC |
| 6 | Ashwagandha | Withania somnifera | Solanaceae | Root | 129mg | Withanolides | Gravimetric |
| 7 | Jasadh bhasma | Zinc | NA | Powder | 20mg | Zinc | UV Visible Spectrophotometry |

HPLC: High performance liquid chromatography
